# Allergic Responses Induced by a Fungal Biopesticide *Metarhizium anisopliae* and House Dust Mite Are Compared in a Mouse Model

**DOI:** 10.1155/2011/360805

**Published:** 2011-06-21

**Authors:** Marsha D. W. Ward, Yong Joo Chung, Lisa B. Copeland, Donald L. Doerfler

**Affiliations:** National Health and Environmental Effects Research Laboratory, U.S. Environmental Protection Agency, 109 T. W. Alexander Drive, MD B105-02, Research Triangle Park, NC 27711, USA

## Abstract

Biopesticides can be effective in controlling their target pest. However, research regarding allergenicity and asthma development is limited. We compared the ability of fungal biopesticide *Metarhizium anisopliae* (MACA) and house dust mite (HDM) extracts to induce allergic responses in BALB/c mice. The extracts were administered by intratracheal aspiration at doubling doses (2.5–80 *μ*g protein) 4X over a four-week period. Three days after the last exposure, serum and bronchoalveolar lavage fluid (BALF) were collected. The extracts' relative allergenicity was evaluated based on response robustness (lowest significant dose response compared to control (0 *μ*g)). MACA induced a more robust serum total IgE response than HDM. However, in the antigen-specific IgE assay, a similar dose of both MACA and HDM was required to achieve the same response level. Our data suggest a threshold dose of MACA for allergy induction and that *M. anisopliae* may be similar to HDM in allergy induction potential.

## 1. Introduction

The toxicity associated with many chemical pesticides has provided the impetus to develop biological agents, either native or genetically engineered, for pest control. Although the microorganisms (bacteria, viruses, and fungi) that have been identified or developed for release in the environment to address problems such as pest control [1–3] can be effective in controlling their target pest, adverse health impacts to mammalian species including humans may be a consequence of exposure to these organisms. Generally, the research regarding mammalian health impacts has focused on the potential toxicity and pathogenicity of these agents. However, research regarding the allergenicity of the agents is more limited. 

Allergy (atopy), a Type I or immediate-type hypersensitivity reaction, is an immune response to otherwise innocuous “nonself” agents (generally proteins) in genetically predisposed individuals. Although most proteins are capable of eliciting immune responses, not all proteins are allergens [4]. Generally, there are no apparent disease symptoms during allergy induction (sensitization). However, some B cells switch production of immunoglobulin (Ig) isotype from IgM to IgE (or IgG1 in guinea pigs) antibodies. In previously sensitized individuals, allergen reexposure/challenge cross-links antigen-specific mast cell/basophil-bound IgE antibodies resulting in the immediate release of preformed mediators (including histamine and prostaglandins). Allergy symptoms become apparent as these mediators produce bronchoconstriction, increased vascular permeability, and inflammation. The clinical manifestations of allergy range from skin rashes and rhinitis to life-threatening asthmatic and anaphylactic reactions. In the case of asthma, a late-phase response may also be seen between 2–12 hours after challenge characterized by mucus hypersecretion, bronchoconstriction, airway hyperresponsiveness (AHR) to nonspecific stimuli, for example, histamine or methacholine (Mch), and eosinophilic airway inflammation. 

The incidence of asthma has increased dramatically over the last several decades in the U.S. and other industrialized nations, particularly for allergic asthma [5, 6]. This trend has been noted in both the general population and in occupational settings [7]. It is assumed that changes in environment, lifestyle, and/or medical practices have contributed to the increase, as the change is more dramatic than would be expected for a simple population genetic shift [8, 9]. Allergic potential is of particular concern because, in addition to the medical and the economic burden on the general population [10], the percentage of adult asthmatics that are allergic asthmatics is estimated to range from 50% [11] to >90% [12]. 

Molds/fungi are ubiquitous in the environment and have been implicated in the etiology of respiratory hypersensitivity diseases such as allergic rhinitis, allergic asthma, allergic bronchopulmonary mycoses, and hypersensitivity pneumonitis [13, 14]. In fact, many fungal genera have been associated with allergic lung disease, but only a few fungi are well studied and even fewer fungal allergens well characterized. A recent review [15] described epidemiological evidence of an association between fungal exposure and asthma severity. Although estimates suggest that 3–10% of people worldwide have fungal allergies [16], Mari et al. [17] found that 19.1% of their allergy study population had positive mold skin prick tests. Another factor that could be contributing to the increased incidence of asthma is childhood exposure to molds [18, 19]. There are limited studies addressing the allergenicity of fungal biopesticides. Furthermore, it is also important to assess the relative allergenicity of these agents by comparing responses to those induced by a well-characterized, indoor allergen such as house dust mite (HDM). 

*Metarhizium anisopliae* is a fungal biopesticide that has been used for agricultural pest control for over a century because of its pathogenicity for a broad range of susceptible insect hosts. This entomopathogenic Deuteromycetes (Fungi imperfecti) fungus is a soil borne fungus found worldwide. *M. anisopliae* has been investigated as a control for sugarcane pests [20, 21] as well as other crop pests and ectoparasites including mites [22–24] and livestock ticks [25]. Currently, *M. anisopliae* is used to control insects such as grasshoppers, termites, and thrips and is licensed in the United States for indoor use in cockroach control. Additionally, *M. anisopliae* has been investigated as a mosquito control to combat malaria by impregnating mosquito netting with viable conidia [26, 27]. The conidia are the environmentally stable portion of the fungal life cycle and a viable reproductive unit. Furthermore, Dr. Ravichandra Potumarthi reported at the 2007 International Conference on Bioengineering and Nanotechnology [28] that his laboratory has used *M. anisopliae* in an energy efficient method to produce biodiesel. These studies indicate a potential increase in opportunities for both indoor residential and occupational exposures to what is ecologically an outdoor organism. 

There is no evidence that *M. anisopliae *is toxic to humans or other mammals. However, there are several case studies documenting infection by *M. anisopliae* in immunocompromised [29] and immunocompetent [30] individuals as well as a case of invasive rhinitis in a cat [31]. Thus suggesting that in some instances humans and other mammals may be susceptible to *M. anisopliae* infection. Previous studies in our laboratory [32, 33] have demonstrated that multiple respiratory exposures to *M. anisopliae* extract can elicit inflammatory and respiratory physiological responses characteristic of human allergic asthma in BALB/c mice. Furthermore, anecdotal information as well as limited clinical data has suggested that some individuals exposed occupationally to *M. anisopliae* have become sensitized [34]. More recently human sera IgE reactivity with *M. anisopliae* has been identified. Barbieri et al. [35] demonstrated positive skin-prick test reactions to *M. anisopliae* in occupationally exposed sugarcane workers with allergic asthma and/or rhinitis. Ward et al. [36] found human serum IgE reactivity with catalase in *M. anisopliae* extract Western blots. However, no IgG reactivity was identified, suggesting that the IgE reactivity was the result of cross-reactivity to catalase from another source. Additionally, Instanes et al. [37] demonstrated that *M. anisopliae* mycelia extract contains substances that have adjuvant activity in a mouse ovalbumin allergy model. These studies indicate that exposure to *M. anisopliae* not only may induce allergic responses but could potentiate allergic responses to other allergenic agents. 

The study goal was to compare the allergic responses induced by the mold extract, *M. anisopliae* (MACA), to those of house dust mite extract (HDM), a known inducer of allergic asthma [38], in an allergic asthma mouse model.

## 2. Materials and Methods

### 2.1. Animals

Fifty-day-old female BALB/c mice (Charles River, Raleigh, NC) were group-housed in polycarbonate cages with hardwood chip bedding in an environmentally controlled, American Association for Accreditation of Laboratory Animal Care-accredited vivarium. Mice were maintained on a 12-h light/dark cycle and allowed access to food (Purina Rodent Lab Chow, St. Louis, MO) and water *ad libitum.* Mice were allowed to acclimate one week prior to the start of the experiment. All animal procedures were reviewed and approved by the Institutional Animal Care and Use Committee of NHEERL, US EPA.

### 2.2. Fungal Antigen Preparation

*M. anisopliae* strain 1080 was obtained from USDA-ARS Entomopathogenic Fungus Collection (Ithaca, NY). The fungus was grown as described in Ward et al. [39]. Briefly, mycelium (hyphae growth) was grown at 27°C for 72 h in Sabouraud's maltose broth with aeration (150 rpm). Subsequently, the mycelium was washed twice with saline to remove media contaminants and air-dried overnight in a biosafety cabinet. Fungal cultures for conidia development were incubated at 27°C on Sabouraud's maltose agar for 2 to 3 weeks. These components were extracted by grinding with a sterile mortar and pestle for approximately 5 min followed by Polytron (Kinematica, Basel, Switzerland) homogenization for 2 min in a total of 15 volumes (by weight) of Hanks' balanced salt solution (HBSS, Gibco BRL, Life Technologies Inc., Rockville, MD) +0.05% (v/v) Tween 80 (Fisher Scientific, Pittsburgh, PA). The resulting suspension was stirred overnight at 4°C, and then centrifuged at 12,500 ×g for 1 h at 4°C. The supernatant was decanted, and then adjusted to pH 6.0 with HCl. Additionally, a deprivation medium (absence of readily available nitrogen and carbon) of unpurified chitin (Sigma Chemical Co., Grand Island, NY) at 3% in water was inoculated with *M. anisopliae *and incubated at 27°C for 72 h with aeration (150 rpm) for higher yields of inducible proteases and chitinases [40, 41]. The filtrate was retained following passage through Whatman no. 1 filter paper. Each filtrate was concentrated with a stirred-cell concentrator (Amicon, Inc., Beverly, MA) using YM3 membranes (molecular weight > 3000 Da cutoff) and filter sterilized with a 0.2 *μ*m syringe filter. Each individual crude antigen extract was assayed for total protein concentration using a Pierce BCA protein assay kit (Pierce, Rockford, IL) with bovine serum albumin as a standard according to manufacturer's procedures. The three component extracts were combined in equal protein amounts to provide *M. anisopliae* crude antigen (MACA) extract. Endotoxin level of the extract was measured using a Limulus amoebocyte lysate test kit (BioWhittaker, Walkersville, MD); the level at the highest dose (80 *μ*g protein) was 0.28 endotoxin unit (EU).

Lyophilized whole body extracts of house dust mites (HDM) *Dermatophagoides farinae* and *D. pteronyssinus* purchased from Greer Laboratories (Lenoir, NC), were rehydrated in HBSS to 3 mg/mL, mixed equally and stored in aliquots at –80°C until use. Endotoxin level of the highest dose (80 *μ*g protein) was 0.27 EU.

### 2.3. Experimental Design

**Figure 1:**
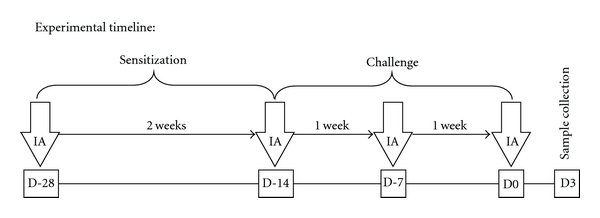
Experimental Timeline. Mice were administered MACA, HDM, or HBSS (control group) by intratracheal aspiration (IA) 4 times over a 4-week period. Allergy sensitization and challenge phases are indicated. Blood and BALF were collected 3 days after the final exposure.

For each protein extract, 6 mice were exposed to 2.5, 5, 10, 20, 40, or 80 *μ*g of extract in HBSS (total volume of 50 *μ*l) by intratracheal aspiration (IA) 4 times over a four-week period, as previously described [32]. Additionally, control mice were exposed to HBSS alone. Briefly, mice were anaesthetized by inhalation of a mixture of 3% isoflurane and 97% oxygen. Antigen extract was deposited into the oropharynx, after which mice inhaled the extract when their noses were gently occluded with a fingertip. Mice were weighed after each exposure and at necropsy. The rationale for the exposure protocol (Figure 1) is based on the immunology paradigm that the primary immune response is approximately 7–14 days following the initial exposure (Day 28 to Day 14, see Figure 1) and approximates the sensitizing phase of allergy induction. The second exposure in our protocol is 14 days (D 14) after the first and thus is on the boundary between sensitization and challenge. However, the last two exposures at D 7 and D 0 are challenge exposures. Serum and bronchoalveolar lavage fluid (BALF) were collected three days following the final exposure. BALF was assessed for lactate dehydrogenase (LDH) activity, total protein, total cell, and differential cell counts. Serum was assessed for total and mold or dust mite-specific IgE. The responses of allergen extract-treated mice were compared to results obtained from HBSS control animals.

### 2.4. Bronchoalveolar Lavage (BAL) and Blood Collection

Blood and BALF samples were collected as previously described in [39]. Briefly, mice were anaesthetized with sodium pentobarbital and blood samples were collected by cardiac puncture. The blood was allowed to clot for 1-2 h at room temperature prior to serum separation by centrifugation, and the serum was stored at –80°C until analysis. The lungs were lavaged twice with 1 mL aliquots of HBSS, and the pooled BALF aliquots were stored on ice. The BALF was centrifuged at 100 ×g for 15 min at 4°C. An aliquot of the supernatant was stored at 4°C for total protein and LDH activity assay and the remainder stored at −80°C. The cell pellet was resuspended in 1 mL of HBSS. Total BALF cell counts were obtained using a Coulter counter (Coulter Corp., Miami, FL). Additionally, 100–150 *μ*L of resuspended cells were adhered onto glass slides at 200 rpm for 10 min using a Cytospin 2 centrifuge (Shandon Inc., Pittsburgh, PA). The BALF cells on glass slides were stained with Wright-Giemsa (Fisher Scientific) on a Hema-Tek 2000 slide stainer (Miles, Inc., Elkhart, IN) and were differentially counted at 200 cells per slide (one slide per animal).

### 2.5. Total Protein and Lactate Dehydrogenase (LDH) Assays

BALF samples were assayed for total protein using Pierce Coomassie Plus Protein Assay Reagent (Pierce). Concentrations were determined from a standard curve using BSA standards obtained from Sigma Chemical Co. (St. Louis, MO). Additionally, the BALF samples were assayed for LDH activity using a commercially prepared kit and controls from Sigma Chemical Co. The assays were modified for use on the KONELAB 30 clinical chemistry spectrophotometer analyzer (Thermo Clinical Labsystems, Espoo, Finland).

### 2.6. Total IgE ELISA

All reagents and incubations were at room temperature, and all volumes added were 100 *μ*L unless otherwise noted. Total IgE ELISA was performed as previously described by Ward et al. [39]. Purified monoclonal antimouse IgE (PharMingen, San Diego, CA) in phosphate buffered saline (PBS), pH 7.3, was used as the capture antibody and biotinylated rat antimouse IgE (BD Bioscience PharMingen) as the detection antibody. Streptavidin-conjugated horseradish peroxidase (Zymed, San Francisco, CA) was the detection enzyme with TMB substrate (DAKO Corporation, Carpinteria, CA). Optical density was read on a Thermomax Plate Reader (Molecular Devices Corp., Menlo Park, CA) at a wavelength of 650 nm. Softmax Pro version 2.6.1 (Molecular Devices Corp.) software was used for data collection and conversion from optical density to protein concentrations. The limit of detection for this assay was 6.25 ng/mL.

### 2.7. Antigen-Specific IgE Assay

A rat basophilic leukemia (RBL) cell beta-hexosaminidase release assay was performed as an indirect measure of antigen-specific IgE in serum. The procedure is based on the method by Hoffmann et al. [42], with modifications by D. Leadbeater and D. A. Basketter (personal communication, Unilever Safety and Environmental Assurance Center, UK). The procedures have been previously described in Chung et al. [43]. Briefly, 96-well flat bottom tissue culture plates were seeded with 10^5^ RBL-2H3 cells (ATCC, Rockville, MD) and incubated for 18 hr at 37°C in a 5% CO_2_ humidified incubator. The cells were passively sensitized with a 1 : 4 dilution of individual mouse serum or with normal sera (spontaneous and total release controls) for 2 hr. Cells were then washed with Tyrode's buffer followed by the addition of allergen extracts (10 *μ*g) and incubated for 1 hr at 37°C in a 5% CO_2_ humidified incubator. For spontaneous release, cells were incubated with buffer alone. Beta-hexosaminidase, the mediator measured, is a chitinase and chitinases are produced by some fungi. Therefore an additional control was used to identify extract-specific (endogenous) chitinase activity; both MACA and HDM extracts were added to cells that were not passively sensitized (no serum). The higher OD for spontaneous release or extract-specific release was used to calculate the % of total mediator release. For total release, cells were incubated with 1% (v/v) Triton X-100 (Sigma) in Tyrode's buffer. Subsequently, the beta-hexosaminidase activity was measured by combining cell culture supernatant and substrate (p-nitrophenyl N-acetyl beta-d-glucosaminide) in a sterile 96-well plate and incubating the plate for 1 hr at room temperature. The enzyme reaction was stopped by the addition of 0.2 M glycine. Absorbance was measured 30 min later using a SpectraMax 340 PC Plate Reader (Molecular Devices Corp.) at a wavelength of 405 nm and data collected by Softmax Pro software (version 2.6.1, Molecular Devices Corp.). Data are presented as percent total release following subtraction of spontaneous release or extract-specific activity if higher. Additionally, the extract dose resulting in 10% of total release was calculated by interpolation.

(1) $$\begin{array}{cc} & \text{\% of Total beta-hexosaminidase Release}\\ & =\frac{\text{Sample Release OD}-\left(\begin{array}{c} \text{Spontaneous Release OD}\\ \text{or}\\ \text{Extract-Specific Release OD} \end{array}\right)}{\text{Total Release OD}-\text{Spontaneous Release OD}}\times 100. \end{array}$$

Additionally, endogenous chitinase activity was measured for both HDM and MACA by adding chitinase substrate to 0, 1, 5, and 10 *μ*g/mL of each extract, as well as, 10 *μ*g/mL of trypsin and processing as described above.

### 2.8. Protease Assay

Extracts were analyzed for protease activity using the EnzChek7Protease Assay Kit (red fluorescence) (Molecular Probes, Eugene, OR). Triplicates of five concentrations (0–20 *μ*g/mL) of the extracts as well as a trypsin enzyme control were added to the digestion buffer (200 mM Tris-HCl, pH 7.8, containing 2 mM sodium azide). Following the addition of BODIPY7 TR-X labeled casein substrate in 0.1 M sodium bicarbonate, pH 8.3, the samples were protected from light and incubated for 1 hr. Protease-catalyzed hydrolysis of the substrate and subsequent release of BODIPY7 TR-X dye-labeled peptides results in increased fluorescence that is proportional to protease activity. Fluorescence of the BODIPY7 TR-X dye was read in a fluorometer (Spectra Max, Gemini XS, Molecular Devices) using a filter with excitation = 590 ± 10 nm, emission = 645 ± 20 nm. Data are reported as percent of trypsin control.

### 2.9. Statistical Analysis

The data were analyzed in two stages. First, a two-way analysis of variance (ANOVA) model was applied to the data, without the control group. This allows the examination of the main effects, of exposures (HDM and *M. anisopliae*) and doses (2.5, 5, 10, 20, 40, 80 *μ*g extract protein), and interactive effects. Additionally, exposure-groups were formed and compared to the control values, using a Dunnett's test. This was done to examine differences from the control group without reference to the ANOVA findings. The level of significance was set at 0.05.

Additionally, the estimated dose at which 10% of total mediator release occurred was calculated for the antigen-specific IgE (RBL assay) data. This was accomplished through linear interpolation between responses at consecutive doses bracketing the 10% value. The level of significance was set at 0.05.

## 3. Results

### 3.1. Change in Body Weight

**Figure 2:**
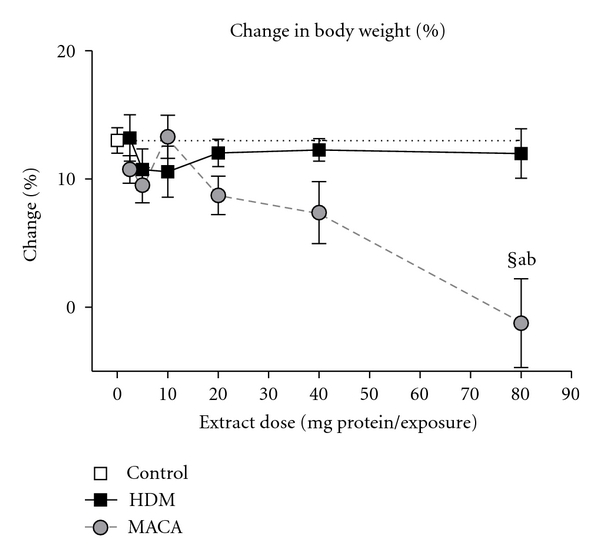
% change in body weight from first exposure to necropsy. (§) the lowest significant dose compared to HBSS (0 dose control), statistically significant differences (a) between extracts at the same dose and (b) from all lower doses within treatment.

Although the mice were weighed after each exposure and at necropsy (3 days after the 4th exposure) to assess overt toxicity of the extracts, the percent weight change was calculated based on their weight after the first exposure and at necropsy. Control mice (HBSS exposures only) increased their body weight by 13.01%  ± 0.99 over the study time course (Figure 2). The percent weight change for the HDM-exposed mice was similar to that of the control mice with the lowest percent weight change being 10.57% ± 1.99 at the 10 *μ*g dose level. MACA-exposed mice tended to have a lower and dose-dependent percentage of weight change than controls, reaching a significant difference at the 80 *μ*g dose level (−1.25% ± 3.46). 

### 3.2. Total Protein and Lactate Dehydrogenase (LDH) Levels

**Figure 3:**
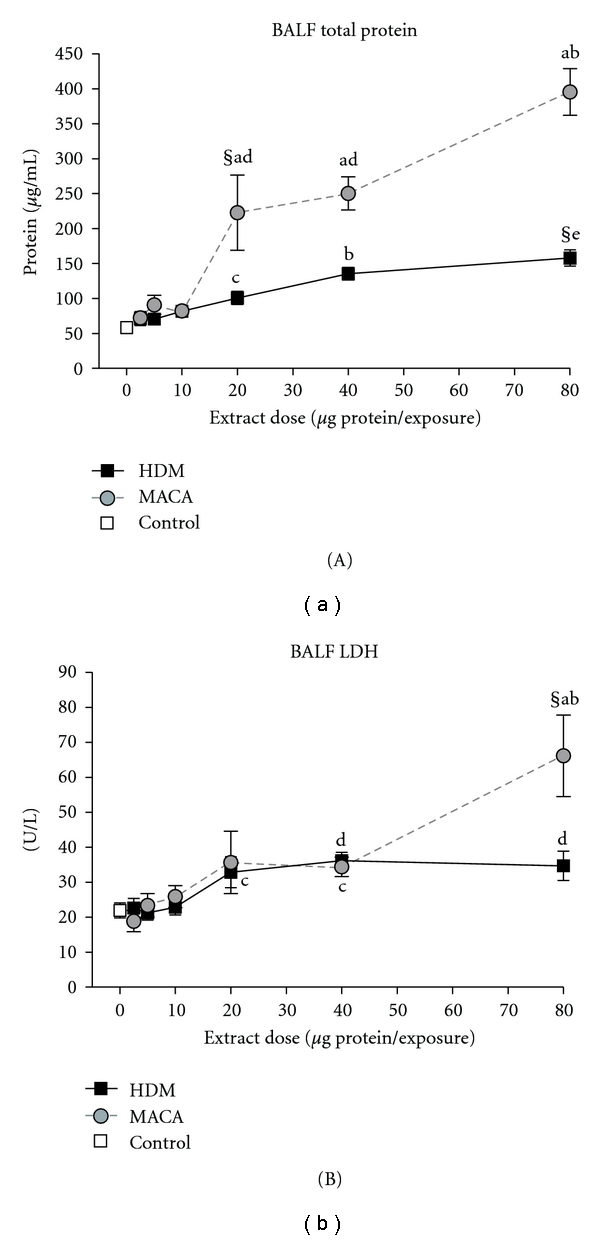
Total protein levels (A), and LDH activity (B) in BALF collected 3 days after the final exposure to HDM or MACA. Symbols indicate (§) the lowest significant dose compared to HBSS (0 dose control), statistically significant differences (a) between extracts at the same dose, (b) from all lower doses within treatment, (c) from 2.5 *μ*g and 5 *μ*g doses within treatment, (d) significantly different from 2.5 to 10 *μ*g doses within treatment, or (e) significantly different from 2.5 to 20 *μ*g doses within treatment (*P* < .05). Error bars represent standard error of the mean. Control *n* = 12; HDM and MACA *n* = 5-6.

Elevations in BALF total protein are indicative of pulmonary vascular leakage (pulmonary edema). Mice exposed to 20 *μ*g of MACA or 40 *μ*g of HDM showed a significant increase in total protein levels compared to the 0 *μ*g dose control mice (Figure 3(A)). The response to HDM and MACA extracts displayed a trend toward dose-dependent increases particularly at high doses. However, mice treated with 20–80 *μ*g of MACA had a significant increase in the total protein level compared to mice exposed to similar doses of HDM.

Elevated LDH activity in BALF is indicative of nonspecific cellular damage. Mice exposed to 80 *μ*g of MACA showed a significant increase in LDH activity compared to the 0 *μ*g dose controls and 80 *μ*g of HDM (Figure 3(B)). In this study, HDM-treated mice did not have significantly different LDH activity at any dose level.

### 3.3. BALF Total and Differential Cell Counts

**Figure 4:**
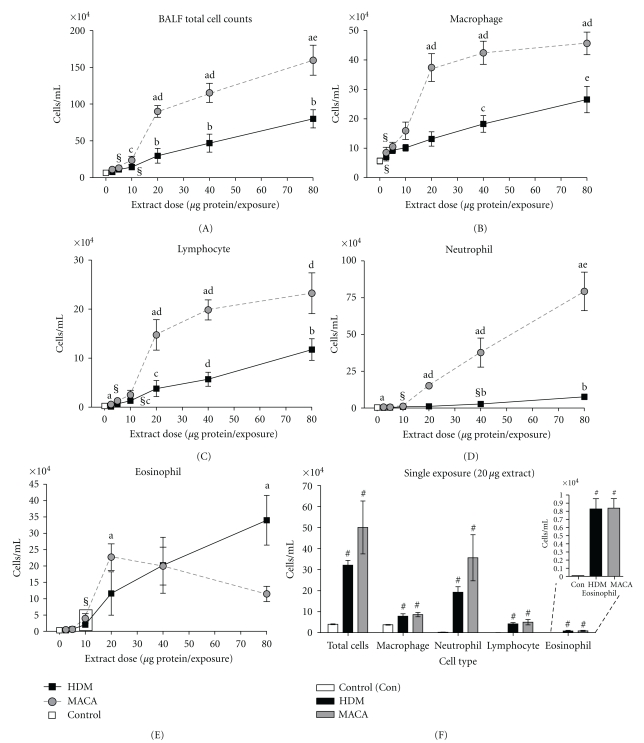
Total cell counts (A) and differential cell counts (Macrophage (B), Lymphocyte (C), Neutrophil (D), and Eosinophil (E)) in BALF of HDM- or MACA-exposed mice collected 3 days after the final exposure. Control *n* = 12; HDM and MACA *n* = 5-6. (F) BALF total and differential cell counts 2 days after a single exposure to HBSS or 20 *μ*g of either HDM or MACA. Control *n* = 4; HDM and MACA *n *= 5. Symbols indicate (§) the lowest significant dose compared to HBSS (0 dose control), statistically significant differences (a) between extracts at the same dose, (b) from all lower doses within treatment, (c) from 2.5 *μ*g and 5 *μ*g doses within treatment, (d) significantly different from 2.5 to 10 *μ*g doses within treatment, (e) significantly different from 2.5 to 20 *μ*g doses within treatment, (#) significantly different from control (*P* < .05). Error bars represent standard error of the mean.

BALF total and differential cell counts were assessed to determine the relative effect of the extracts in the development of the late-phase allergic response in this mouse model. BALF total cell counts were dose dependently increased in HDM- and MACA-exposed mice (Figure 4(A)). The lowest doses of MACA and HDM that induced a significant increase in total cell counts compared to 0 *μ*g dose controls were 5 *μ*g (12.97 ± 2.15 × 10^4^ cells/mL) and 10 *μ*g (14.28 ± 1.73 × 10^4^ cells/mL), respectively.

Both BALF macrophage and lymphocyte counts were dose dependently increased in HDM-exposed mice across the dose range. MACA-exposed mice demonstrated dose-dependent increases in both cell types through the 20 *μ*g dose level, then the responses plateaued at much higher levels than those of HDM-exposed mice (Figures 4(B) and 4(C)). The lowest doses of MACA and HDM that caused a significant elevation in macrophage counts compared to 0 *μ*g dose controls were 2.5 *μ*g (8.48 ± 1.80 × 10^4^ cells/mL) and 5 *μ*g (9.30 ± 1.15 × 10^4^ cells/mL), respectively (Figure 4(B)). The lowest doses of MACA and HDM that induced a significant increase in lymphocyte counts compared to 0 *μ*g dose controls were 5 *μ*g (1.30 ± 0.61 × 10^4^ cells/mL) and 10 *μ*g (1.33 + 0.21 × 10^4^ cells/mL), respectively (Figure 4(C)). 

Dose-dependent increases in BALF neutrophil counts were observed in HDM- and MACA-treated mice (Figure 4(D)). The lowest doses of MACA and HDM that caused a significant elevation of neutrophil counts compared to 0 *μ*g dose controls were 10 *μ*g (1.16 + 0.38 × 10^4^ cells/mL) and 40 *μ*g (2.72 + 0.69 × 10^4^ cells/mL), respectively. 

Mice exposed to 20–80 *μ*g of MACA showed significant increases in total cell, macrophage, and neutrophil cell counts. A similar pattern of response was demonstrated by lymphocyte counts; however, at the 80 *μ*g dose the differences between the treatments were not significant.

Eosinophilic airway inflammation is an endpoint characteristic of allergic asthma responses. BALF eosinophil counts were increased in a dose-dependent matter through the dose range for HDM and in the lower dose range for MACA. The lowest dose that induced a significant elevation of eosinophil counts compared to 0 *μ*g dose controls was 10 *μ*g for both HDM and MACA (Figure 4(E)). HDM-treated mice showed increasing eosinophil counts to the highest dose (80 *μ*g; 33.98 ± 7.61 × 10^4^ cells/mL). However, MACA-treated mice showed a maximum number of eosinophils at 20 *μ*g (22.72 ± 4.04 × 10^4^ cells/mL) dose. Although the eosinophil numbers tended to decline with the increase in dose levels, this decline in MACA-induced eosinophil influx were not statistically significant. There was no statistically significant differences the eosinophil influx induced by the two extracts except at the 80 *μ*g dose level where HDM-induced eosinophil counts were significantly higher than those for MACA-exposed mice. Whether the limited weight gain at higher doses is related to the MACA-induced eosinophil influx decline with increases in dose is unclear.

In a separate study, mouse BALF was collected 2 days after the mice received a single 20 *μ*g dose of extract. Both HDM and MACA were able to induce a robust influx of neutrophils, as well as, a small but significant influx of eosinophils compared to control mouse responses (Figure 4(F)). However, as expected, there was no significant difference in the serum total or antigen-specific IgE responses induced by either extract compared to control mice 2 days after a single extract exposure (data not shown).

### 3.4. Total and Antigen-Specific IgE

**Figure 5:**
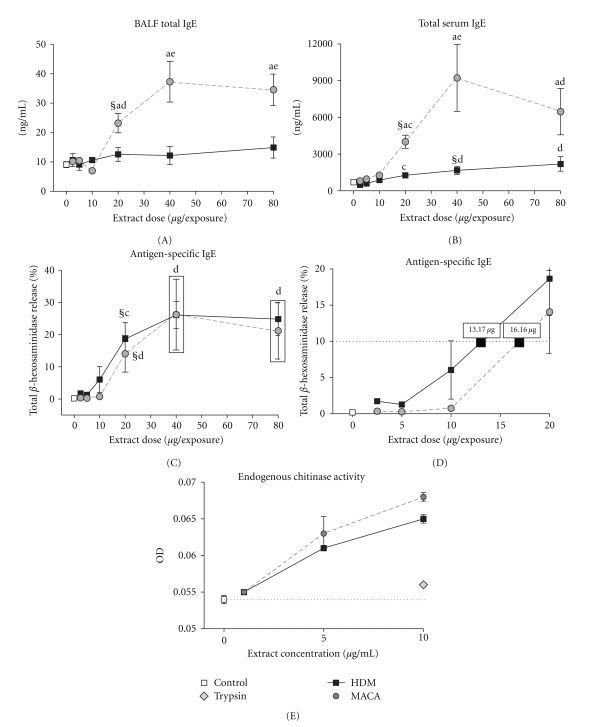
BALF (A) and Serum (B) total IgE levels of HDM- or MACA-exposed mice collected 3 days after the final exposure. (C) Antigen-specific IgE indicated by % of RBL cells total beta-hexosaminidase release across the dose range after incubation with serum (1 : 4 dilution) obtained from HDM- or MACA-exposed mice. (D) A detail of the antigen-specific IgE dose-response displaying the extract doses calculated to result in 10% of total beta-hexosaminidase release (dashed line). (E) The endogenous chitinase activity in the MACA and HDM extracts. Symbols indicate (§) the lowest significant dose compared to HBSS (0 dose control), statistically significant differences (a) between extracts at the same dose, (b) from all lower doses within treatment, (c) from 2.5 *μ*g and 5 *μ*g doses within treatment, (d) significantly different from 2.5 to 10 *μ*g doses within treatment, (e) significantly different from 2.5 to 20 *μ*g doses within treatment (*P* < .05). Error bars represent standard error of the mean. Control *n* = 12; HDM and MACA *n* = 5-6.

Elevated IgE levels are one of the key indicators of atopic status and are frequently associated with allergic airway disease. BALF total IgE increased in MACA-treated mice dose dependently. The 20 *μ*g dose was the lowest significant dose compared to the 0 *μ*g control mice (Figure 5(A)). Additionally, MACA induced significantly more BALF total IgE than HDM from across the 20–80 *μ*g dose range. HDM-treated mice did not demonstrate a significant increase in BALF total IgE at any dose. 

MACA-treated mice demonstrated dose-dependent increases in serum total IgE up to the 40 *μ*g dose level but tended to decline at the 80 *μ*g dose level. Although HDM-treated mice demonstrated a dose-dependent increase in serum total IgE throughout the dose range the magnitude of the response was significantly lower than that induced by MACA at the 20–80 *μ*g doses (Figure 5(B)). The lowest dose of MACA or HDM that induced a significant increase in total IgE compared to 0 *μ*g dose controls was 20 *μ*g and 40 *μ*g of extract, respectively. 

As an indirect measure of functional antigen-specific IgE, the release of the preformed mediator beta-hexosaminidase was measured using the rat basophil leukemia (RBL) cell assay. When analyzed across the dose range, it required 20 *μ*g of extract to induce a significant increase compared to the 0 *μ*g dose control for both MACA and HDM (Figure 5(C)). To provide a basis for comparing the extracts' ability to induce antigen-specific IgE, the extract dose resulting in 10% of total beta-hexosaminidase release was calculated (Figure 5(D)). This calculated dose indicated that it required slightly less HDM extract (13.17 *μ*g) to achieve 10% of total mediator release than MACA (16.16 *μ*g). 

The mediator beta-hexosaminidase measured in the RBL assay is a chitinase. Therefore, it was important to determine if any portion of the mediator release measured in that assay was due to endogenous levels of chitinase activity (Figure 5(E)). Both MACA and HDM demonstrated similar enzymatic activity which increased with the increase in extract concentration.

### 3.5. Protease Activity

**Figure 6:**
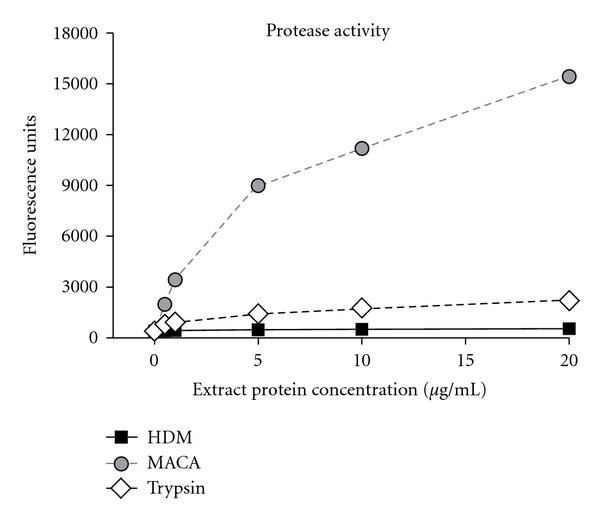
Extract protease activity compared to trypsin protease activity control.

Fungal extract protease activity can amplify the inflammatory responses of allergic disease through protease-activated receptors on epithelial cells [44]. The extracts were assayed for proteolytic activity to investigate how differences might impact extract-induced allergic responses. HDM protease activity ranged from 1/4 to 1/2 of trypsin proteolytic activity. On the other hand, MACA proteolytic activity was more than 6-fold greater than trypsin activity for 5–20 *μ*g/mL extract concentrations (Figure 6). 

## 4. Discussion

Previous studies in our laboratory [32, 33, 39] have found that *M. anisopliae* extract has the capacity to induce allergic responses in a mouse model. In the present study, the relative allergenicity of the biopesticide *M. anisopliae* was compared to HDM induced responses based on various endpoints with an emphasis on those that are characteristic of human allergic responses, for example, serum total and antigen-specific IgE and inflammatory cell (neutrophils and eosinophils) influx into the lungs (summarized in Table 1). 

It is of interest that at higher doses (20–80 *μ*g), MACA-treated mice demonstrated a significantly higher level of serum total IgE than the HDM-treated mice displayed. However, similar doses of each extract were required to induce the same response level (10% of total mediator release) for antigen-specific IgE. We have seen a similar effect in serum total IgE in studies where *Stachybotrys chartarum* was compared to HDM except more *S. chartarum* extract was required to achieve the same response level in the antigen-specific IgE assay [45]. This “by-stander” effect was demonstrated by Moss [46] who found an IL-4-dependent elevation of nonspecific IgE was induced by *A. fumigatus *in an allergic bronchopulmonary aspergillosis mouse model.

Although a single exposure to either of these extracts (20 *μ*g) did not induce increases in serum or BALF total IgE or antigen-specific/functional IgE (serum), it did result in a significant increase in BALF total and differential cells compared to controls (Figure 4(F)) but to varying degrees. There were a prominent increase in neutrophils and a small but significant increase in eosinophils compared to the controls. The ability to induce this neutrophilic response in naïve individuals could enhance their potential to induce and/or exacerbate allergic asthma [47, 48]. A direct comparison of the single and multiple exposure results cannot be made since these were performed in separate studies. However, it appears that multiple exposures may have a damping effect on the neutrophil influx suggesting the impact on allergy might reside in early exposures but does not preclude the potential to exacerbate allergic asthma symptoms. 

An unexpected finding was the decline in percent body weight change in MACA-exposed mice with the increase in exposure dose compared to both control and HDM-exposed mice. The necropsy weight was measured 3 days after dosing; this could reflect a general malaise induced for a short period following MACA dosing. However, when percent weight change was calculated using mouse weight at the 4th exposure (7 days after the previous dose) a similar but less dramatic pattern of weight change existed (data not shown). We have also observed a less dramatic decline in percent body weight with mice exposed to high doses of *Stachybotrys chartarum* extract (unpublished data). These data suggest the possibility of overt toxicity at higher exposure doses of some molds and the need for further investigation in the area.

There are a number of factors that might affect the differential responses seen between the two extracts studied. One such factor might be differences in the quality or quantity of the “allergens” among the extracts. As was shown in murine model of allergic asthma,* A. fumigatus* allergens differ in their ability to induce IgE, eosinophils, and airway hyperresponsiveness and can differentially affect the allergic asthma pathogenesis [49]. Furthermore, the authors found that in addition to the allergens, other extract components such as enzymes and toxins contributed to the overall responses leading to allergic asthma. Although allergens have been identified in *M. anisopliae* [35, 36], possible differences in allergen quality or quantity among molds have not been described. 

Protease activity has been shown to play a role in allergic responsiveness [50] possibly by causing epithelial cell damage [51] or by the amplification of allergic inflammatory responses through protease-activated receptors on epithelial cells [44]. MACA had significantly more protease activity than the trypsin control or HDM which were similar. Furthermore, in this study a lower dose of MACA versus HDM was required to induce a significant response increase compared to control animals. Therefore, protease activity may have had some impact on allergy induction. 

Chitinases are widely distributed in living organisms and are thought to primarily play a role in pathogen defense. Recently, elevated levels of acid mammalian chitinase were observed in the lung tissue of asthmatic patients [52], and exogenous chitinase (*Streptomyces griseus)* was shown to activate Protease-activated Receptor-2 in human airway epithelial cells [53]. Chitinases have been found in *M. anisopliae *[41, 54]. Although chitinase activity for both extracts was elevated compared to the protease (trypsin) control and the background control, it is not clear from our current study what impact this may have had on allergy development. This area merits further examination. 

Coexposure to endotoxin is another factor that may influence allergic responsiveness. In our study, the *M. anisopliae* and HDM extracts had the highest and similar levels of endotoxin (0.28 EU and 0.27 EU in 80 *μ*g of extract protein) which equate to ~0.028–0.056 and 0.027–0.054 ng LPS, respectively. This is well below the levels expected to have an impact on allergic responses [55–58] suggesting that the endotoxin levels in these extracts had little if any effect on the response outcome in this study. 

Exposure assessment and threshold sensitizing dose are critical in understanding the development of allergic disease with regard to a given agent. Studies have found that an exposure to >2 *μ*g* Der p* 1/g dust is a risk factor for HDM sensitization in atopic children [59, 60] but 80 *μ*g *Der* group 1 allergen/g dust was required to sensitize nonatopic children [61]. Additionally, Arbes et al. [62] estimated that most beds in US homes have detectable levels of HDM with 2 *μ*g HDM group 1 allergen/g dust in 46.2% ± 2% of beds and 10 *μ*g/g dust in 24.2% ± 2.1% of beds. Even though dust allergen load and aspiration exposures (as in the current study) are not equivalent, the fact that there were 1.96 *μ*g of *Der* group 1 allergens in 10 *μ*g HDM extract protein suggests that dosing in our study was within the range of human exposure. 

The sensitizing exposure threshold dose like the one defined for HDM is not available for most allergens including molds. No exposure studies were found for *M. anisopliae*. Dose-response comparisons like the one presented in this study may provide insight into sensitizing dose thresholds. The data suggest that *Metarhizium anisopliae* may be similar to HDM in allergenic potency. However, it must be noted that the allergen load in the *M. anisopliae* extract is unknown. Furthermore, the HDM extract used in this study is lyophilized whole bodies, that is, not including fecal matter which has a higher concentration of allergens. Therefore, the allergen load in the HDM extract might be lower than would be seen in a typical human exposure. 

A variety of biopesticides that are available have been vetted for toxicity and pathogenicity. However, there is limited data available regarding their allergenicity. One agent *Bacillius thuringiensis*, in use for many years, has been shown to induce IgE responses from occupational exposures [63, 64]. Additionally, there are both human and animal studies indicating that *M. anisopliae* exposure may induce allergy and possibly asthma [32, 33, 35]. Although the data in this report suggests that *M. anisopliae* is a robust allergen source, this is not the case for all molds or fungal biopesticides. The evaluation of *Trichoderma viride*, a fungal biopesticide used to treat seed and soil to inhibit other fungal pathogens, demonstrated limited capacity to induce allergic responses compared to HDM in our mouse model (unpublished data). 

At this time, human exposure levels and sensitization thresholds are unknown for most allergens including mold/fungal biopesticides. These critical factors must be considered in evaluating the risk in human allergic disease development. Importantly, occupational and residential exposures may increase with an increased use of biological agents or their products (metabolites, enzymes) as biopesticides. Although human exposure levels are not within the scope of this paper, the data presented in this study suggest a threshold dose of M. anisopliae for the induction of allergic responsiveness which may play a role in asthma development.

## Figures and Tables

**Table 1:** Summary of endpoints based on the lowest dose that induced a significant response compared to the 0 *μ*g dose^a^ and the magnitude of the response at that dose.

Source	Endpoint	Relative potency		
		Lowest dose for significant response^a^ (response magnitude)		
		MACA		HDM
BALF	Total protein (*μ*g/mL)	20 (222.74 ± 53.81)	≫	80 (57.85 ± 11.69)
	LDH activity (U/l)	80 (66.14 ± 11.66)	≫	NS^b^
	Total cell count (×10^4^ cells/mL)	5 (12.97 ± 2.15)	>	10 (14.28 ± 1.73)
	Macrophage count (×10^4^ cells/mL)	2.5 (8.48 ± 1.80)	>	5 (9.30 ± 1.15)
	Neutrophil count (×10^4^ cells/mL)	10 (1.16 + 0.38)	≫	40 (2.72 + 0.69)
	Eosinophil count (×10^4^ cells/mL)	10 (3.94 ± 1.44)	=	10 (2.02 ± 0.42)
	Lymphocyte count (×10^4^ cells/mL)	5 (1.30 ± 0.61)	>	10 (1.33 ± 0.21)
	Total IgE (ng/mL)	20 (23.21 ± 3.30)	≫	NS

Serum	Total IgE (ng/mL)	20 (4056.98 ± 564.31)	>	40 (1693.79 ± 329.84)
	Antigen-specific Release^c^ (% Total mediator release)	20 (14.06 ± 5.71)	*∼*	20 (18.64 ± 5.08)
	Antigen-specific IgE^d^ (*μ*g extract)	16.16	*∼*	13.17

^a^ Dose (*μ*g extract) resulting in a significant increase over 0 *μ*g dose control at *P* < .01.

^b^ NS: no significant dose compared to 0 *μ*g dose control.

^c^ For antigen-specific IgE, dose comparison was made to the 2.5 *μ*g dose.

^d^ Calculated dose that would induce sufficient antigen-specific IgE resulting in 10% of total mediator release in RBL assay.

## Abbreviations

BALF: Bronchoalveolar lavage fluid
ELISA: Enzyme-linked immunosorbent assay
HBSS: Hank's balanced salt solution
IA: Intratracheal aspiration
IgE: Immunoglobulin E
IgG: Immunoglobulin G
PBS: Phosphate buffered saline
MACA: *Metarhizium anisopliae* crude antigen
HDM: House dust mite
LDH: Lactate dehydrogenase
RBL: Rat basophilic leukemia
IOM: The Institute of Medicine.
